# The Semantics of Priapism and the First Sign of Chronic Myeloid Leukemia

**DOI:** 10.1155/2017/2656203

**Published:** 2017-05-30

**Authors:** Michael R. Minckler, Ellie Conser, Javier J. Figueroa, Aaron J. Scott, Joshua Gaither, Richard Amini

**Affiliations:** ^1^Department of Emergency Medicine, University of Arizona, Tucson, AZ, USA; ^2^College of Medicine, University of Arizona, Tucson, AZ, USA; ^3^Department of Medicine, Division of Hematology and Oncology, University of Arizona Cancer Center, Tucson, AZ, USA

## Abstract

Priapism is defined as an erection that persists beyond four hours, lasting beyond or unrelated to sexual stimulation (Salonia et al., 2014). Because the risk of ischemic damage and impotence is high with priapism (35%), management guidelines are directed towards rapid treatment of this condition (Salonia et al., 2014). This report describes the rare case of an 18-year-old male who presented to the Emergency Department (ED) three times with recurrent and worsening episodes of sustained penile erections. On the patient's third visit, he presented with priapism of greater than six-hour duration that was found to be the result of chronic myeloid leukemia. Clinician awareness of the diagnostic semantics and differential diagnosis surrounding priapism is pivotal in its urgent management.

## 1. Introduction

Priapism is an uncommon complaint for patients presenting to the ED, but it is associated with a 35% risk of impotence [1]. It is defined as an erection that persists beyond four hours, lasting beyond or unrelated to sexual stimulation [2]. Although priapism can be the result of underlying systemic pathology, in the vast majority of cases, the etiology of priapism is already known. The most common causes of priapism include medication side effect, sickle-cell disease, and neurologic disorders [3]. Because the etiology is usually known, management guidelines are generally directed at rapid treatment versus rapid diagnosis, and it is uncommon for clinicians to workup the underlying cause [2].

## 2. Case Report

An 18-year-old previously healthy man presented to the ED with persistent priapism. The patient stated that during the past three months he had been experiencing numerous episodes of priapism unrelated to sexual stimulation that resolved with cold showers. This was the patient's third episode of priapism requiring an ED visit in three months. In October, the patient visited an ED where he was treated with pseudoephedrine and an ice pack. In November, the patient was given terbutaline without success. During that visit, a corporal blood gas was obtained revealing a pH of 7.12, PCO2 of 30, and PO2 of 43, suggestive of ischemic priapism. Of note, the patient's blood gas hemoglobin level returned as “results incalculable.” Due to concern for ischemia, Urology was consulted, and they performed penile aspiration and irrigation.

Upon the patient's third presentation to the ED in December, he had been experiencing symptoms for at least six hours. The patient had no other reported medical conditions, medication list, or symptoms other than three months of intermittent priapism. Due to the patient's prior history of failed medical management of priapism, the Urology service was again consulted who ordered a penile blood gas and performed a penile aspiration and irrigation. Due to the rare presentation and unknown cause for the patient's recurring condition, laboratory blood testing was ordered by the emergency physicians.

A complete blood count (CBC) demonstrated a markedly elevated white blood cell (WBC) count of 588 × 1000/*μ*L with the following differential: absolute neutrophils 264.60 × 1000/*μ*L (normal range (NR) 1.80–8.00 × 1000/*μ*L), absolute lymphocytes 17.64 × 1000/*μ*L (NR 1.20–5.20 × 1000/*μ*L), absolute monocytes 11.76 × 1000/*μ*L (NR 0.30–1.00 × 1000/*μ*L), absolute eosinophils 11.76 × 1000/*μ*L (NR 0.00–0.50 × 1000/*μ*L), absolute basophils 5.88 × 1000/*μ*L (NR 0.00–0.10 × 1000/*μ*L), absolute myelocytes 164.64 × 1000/*μ*L (NR 0.00 × 1000/*μ*L), absolute metamyelocytes 35.28 × 1000/*μ*L (NR 0.00 × 1000/*μ*L), absolute promyelocytes 64.68 × 1000/*μ*L (NR 0.00 × 1000/*μ*L), and peripheral blasts 11.76 × 1000/*μ*L (NR 0.00 × 1000/*μ*L). The CBC also revealed a hemoglobin of 7.3 g/dL and a platelet count of 109 × 1000/*μ*L.

**Figure 1:**
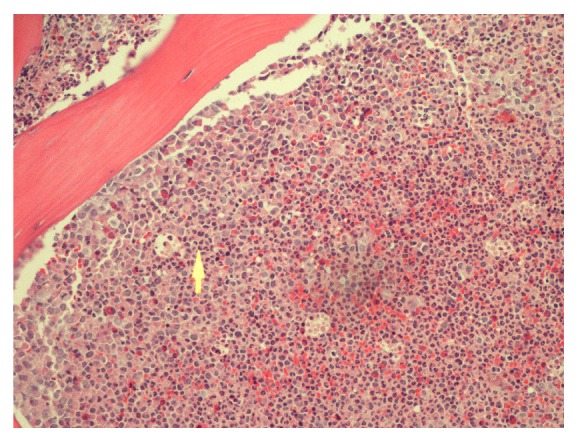
The bone marrow core biopsy H&E stain above shows a hypercellular marrow with 100% cellularity and an increase in granulocyte precursors. In addition, an increase in basophils, eosinophils, and an occasional monocyte and blast are also observed. The arrow points to granulocytes comprising most of the marrow.

**Figure 2:**
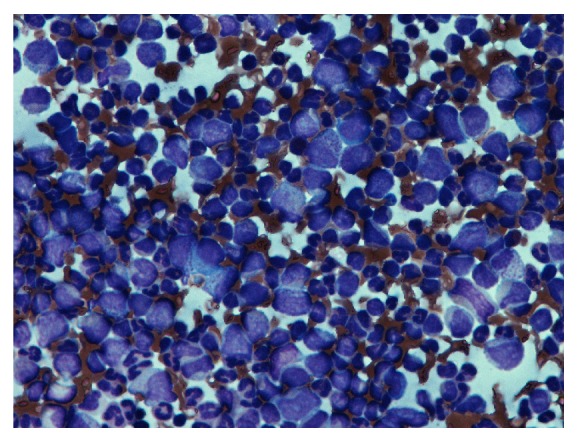
The bone marrow aspirate shown above demonstrates a “left-shift” in granulopoiesis, especially with a significant increase in myelocytes, promyelocytes, and metamyelocytes.

**Figure 3:**
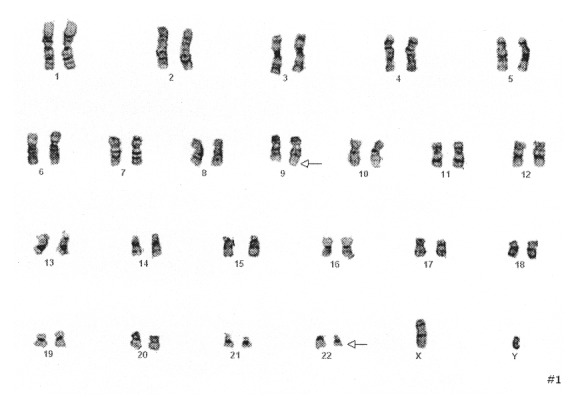
Karyotype analysis obtained from the bone marrow aspirate with the arrow pointing to the t(9;22) translocation.

The Pediatric Hematology and Oncology service was consulted and the patient was admitted to the Pediatric Intensive Care Unit. Given concern for a hematologic malignancy presenting as a vasoocclusive event with elevated peripheral blasts, the patient was started on hydroxyurea. Further workup included a bone marrow aspiration and biopsy with cytogenetic and fluorescence in situ hybridization (FISH) studies. Hematopathology review of the peripheral blood revealed a hyperleukocytosis with absolute neutrophilia and a peripheral blast count of 2%. Review of the bone marrow aspirate and biopsy showed a hypercellular marrow with 4% blasts (see Figures 1 and 2). Chromosomal analysis with cytogenetic karyotyping revealed a t(9;22) translocation (see Figure 3). FISH analysis also confirmed a t(9;22) translocation. The patient was diagnosed with chronic myeloid leukemia (CML) in chronic phase and was transitioned to imatinib 400 mg daily during his admission. He tolerated treatment initiation well without further complications during his hospital stay and he was subsequently discharged home.

## 3. Discussion

Over 95% of cases of priapism are ischemic in nature. High flow arterial type is rare and generally occurs after blunt perineal trauma of sufficient mechanism to cause the formation of an AV fistula. Diagnostically, ischemic priapism will have a PO2 < 30 mmHg, PCO2 > 60 mmHg, and a pH < 7.25. High flow type will be consistent with arterial or normal mixed venous blood with a pO2 > 40 mmHg, pCO2 < 40–50 mmHg, and pH 7.35–7.40. Stuttering priapism is a condition known for recurrent short-lived episodes of erections lasting less than three hours in duration. Stuttering priapism is the least studied form of erections and is commonly associated with sickle-cell ischemic priapism. These patients may present reporting multiple self-limiting episodes of prolonged erection at home' or as in our case, numerous visits to emergency departments [2].

Priapism is a rare presentation to the Emergency Department and as a result management guidelines are largely based on case reports. The guidelines that exist place the major focus on differentiating ischemic versus nonischemic priapism, and delivering rapid therapy in the ED for the management of ischemic penile compartment-syndrome. However, as for the importance of expanded screening for new onset, undifferentiated ischemic pripaism is often forgone as emergency physicians attempt to expedite therapeutic management in an effort to reduce complication rates [4].

The formal definitions of priapism, used by both the American and European Urologic societies, define it as a prolonged erection lasting equal to or greater than four hours. Given the readily available nature of laboratories in the developed world, the formal four-hour minimum should not be used as definitive criteria for diagnosing this condition in the ED. Rather, priapism should be considered in a patient who self-reports prolonged erection, of any length, including stuttering symptoms. The benefit of this is twofold: first, diagnosis of previously unidentified and treatable systemic pathologies such as CML is less likely to be missed. Second, treatment of a causative disease will reduce recurrence rates and the burden of permanent damage to penile tissue. The expanded screening of patients is not complex and, in addition to a penile blood gas, includes a complete blood count with differential and coagulation profile [2]. Additionally, a complete metabolic panel, blood-smear, and other hematologic screening should be considered, if clinical suspicion exists.

Chronic myeloid leukemia (CML) is an indolent form of leukemia of the myeloid lineage and may exist in 3 distinct phases, (1) chronic phase, (2) accelerated phase, and (3) blast crisis [5]. The genetic translocation responsible for this disease is a reciprocal translocation between chromosomes 9 and 22, also known as the Philadelphia chromosome, creating the fusion gene BCR-ABL1 that leads to a constitutively active kinase that drives oncogenesis [5, 6]. Depending on the CML phase and biology, patients may have a profoundly elevated WBC at diagnosis, which can present as leukostasis, or hyperviscosity syndrome. Hyperviscosity syndrome is associated with serious clinical manifestations including visual discomfort (retinopathy, retinal hemorrhages), shortness of breath (pulmonary insufficiency), priapism, and other symptoms related to end organ damage [5, 7]. If there is clinical evidence consistent with hyperviscosity syndrome, emergent therapy and in rare cases leukophoresis can be initiated.

ED management of CML with marked leukocytosis involves intravascular repletion, end organ assessment including lab analysis and oxygen saturation status, and early hematology/oncology consult for aid in diagnostic workup and medical management [1, 8]. Even if a patient is not in acute distress, hospital admission for monitoring by the hematology/oncology service is warranted as these patients are at high risk for acute vascular crisis. Long-term therapy involves daily TKI with the goal of maintaining remission [9].

## 4. Conclusion

Priapism is a condition with a high complication rate whose management is generally focused on rapid treatment. Previously undifferentiated priapism may be the first sign of serious systemic illness. Given the paucity of peer reviewed evidence regarding this condition, we advocate for hematologic screening of these patients based solely on self-reported prolonged erection, regardless of whether or not it lasted for greater than the four hours as stipulated by its current definition.
